# Rehabilitating a Complex Mid-facial Defect With an Interim, Magnet-Retained, Two-Piece Acrylic Prosthesis: A Case Report

**DOI:** 10.7759/cureus.82135

**Published:** 2025-04-12

**Authors:** Suja Joseph, Nazia Rasheed, Angel M Joseph, Shibi Mathew, Joshy P Abraham

**Affiliations:** 1 Prosthodontics, Travancore Dental College, Kollam, IND; 2 General Dentistry and Prosthodontics, Dr. Nazia’s Dental Care, Kottayam, IND; 3 Restorative Dentistry and Prosthodontics, College of Dentistry, Majmaah University, Al Majma’ah, SAU; 4 Prosthodontics, Pushpagiri College of Dental Sciences, Thiruvalla, IND

**Keywords:** carcinoma, facial injuries, magnetics, maxillofacial prosthesis, midfacial defects, oral neoplasms

## Abstract

Mid-facial defects, which often communicate with intraoral maxillary defects, pose significant challenges for prosthetic rehabilitation. An 80-year-old male reported for orofacial rehabilitation following multiple resections for recurrent adenoid cystic carcinoma. The chief concerns include facial appearance, mastication, speech, swallowing, nasal regurgitation, and oral fluid leakage. Examination revealed a complex left-sided mid-facial defect involving the maxilla, infratemporal fossa, and lateral nasal wall, with an intraoral-external communication. Complications included fibrotic tissue healing, trismus, and mandibular deviation. Surgical reconstruction was contraindicated. An interim, magnet-retained, two-piece acrylic prosthesis was fabricated with a palatal obturator without artificial teeth for the intraoral defect and a cheek prosthesis for the external defect. The cheek prosthesis improved facial appearance, while the palatal obturator sealed the intraoral defect, enhancing swallowing and preventing nasal regurgitation and oral fluid leakage; however, mastication and speech were affected. The patient continued with a semi-solid diet and was able to masticate using the remaining dentition. The palatal obturator slightly improved speech by sealing the defect, although slurring persisted. The combined anatomical support and magnetic retention enhanced the retention, stability, and support of the prosthesis. This non-invasive and cost-effective solution was tailored to meet the patient’s specific needs and limitations.

## Introduction

Oral cancer is the most prevalent type of cancer in India, with an age-standardized incidence rate of 12.6 per 100,000 population [1]. This has led to an increase in oncosurgical interventions, often resulting in maxillofacial defects, including mid-facial defects. Mid-facial defects may also arise from acquired causes such as trauma, burns, and conditions like lethal midline granuloma, or from congenital anomalies such as vascular malformations [2]. Marunick et al. have classified these defects into mid-line and lateral-midline defects. Mid-line defects affect the nose or upper lip, creating a connection to the intraoral maxillary defect, while lateral-mid-line defects involve the cheek or orbital area, also communicating with the maxilla [3].

Mid-facial defects, which often communicate with intraoral maxillary defects, pose significant challenges for prosthetic rehabilitation [4-7]. This case describes the rehabilitation of a complex mid-facial defect using an interim, magnet-retained, two-piece acrylic prosthesis, which satisfactorily addressed most of the patient’s chief concerns. Institutional ethical approval was not required for reporting individual cases. Informed consent was obtained from the patient.

This case was presented at the 12th Biennial Congress of the Asian Academy of Prosthodontics, held virtually in Indonesia in August 2021. It was previously posted to the Authorea preprint server on December 2, 2024 with DOI: 10.22541/au.173310679.91733221/v1.

## Case presentation

**Figure 1:**
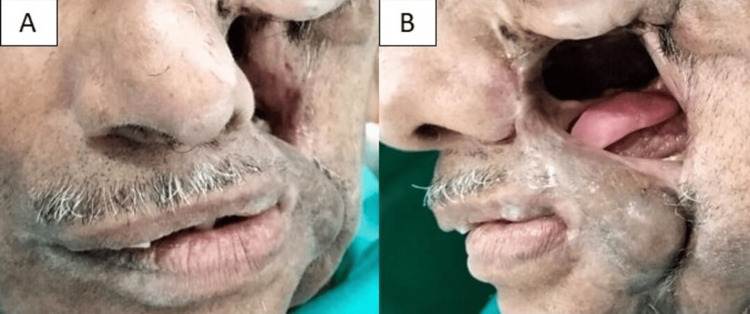
Extraoral views of the mid-facial defect. (A) Extraoral frontal view and (B) lateral view of the defect.

An 80-year-old male reported for orofacial rehabilitation following multiple resections for recurrent adenoid cystic carcinoma and failed tissue reconstruction using pedicle grafts over two years. The chief concerns included facial appearance, mastication, speech, swallowing, nasal regurgitation, and oral fluid leakage. Examination revealed a complex left-sided mid-facial defect involving the maxilla, infratemporal fossa, and lateral nasal wall, with an intraoral-external communication (Figure 1).

The mandibular dentition was preserved, while the maxilla retained teeth 11, 12, and 13. The intraoral findings were confirmed with an orthopantomogram, revealing bone contour and assessing remaining teeth. Complications included fibrotic tissue healing, trismus, and mandibular deviation. The patient presented with an inter-arch space measuring 15 mm in the anterior region. There was no previous prosthesis and the patient was relying on a semi-solid diet. The case was discussed with the oncologist and the onco-surgeon. Surgical reconstruction was contraindicated due to advanced age, compromised medical condition, and potential cancer recurrence.

Mouth-opening exercises and a screw gag prosthesis were prescribed to facilitate the fabrication of a maxillary removable partial denture. However, no significant improvement was observed after a month, possibly due to the complications and delayed prosthetic rehabilitation. Prosthetic rehabilitation was planned using an interim, magnet-retained, two-piece acrylic prosthesis with a palatal obturator without artificial teeth for the intraoral defect and a cheek prosthesis for the external defect. The treatment procedures were as follows.

Impressions and working casts

**Figure 2:**
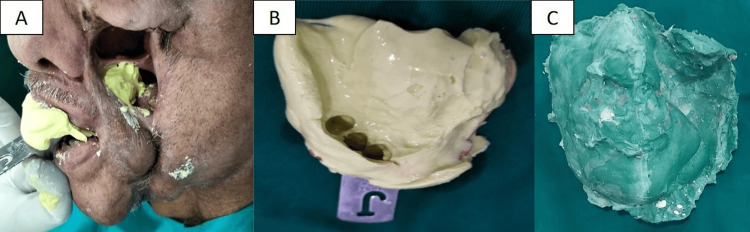
Impression of the intraoral maxillary defect and working cast of the external mid-facial defect. (A) Impression making procedure for the intraoral defect. (B) Impression of the maxilla and intraoral defect. (C) Working cast of the face and external defect.

To obtain impressions, moist gauze was used to prevent the flow of the impression material into undesired areas. The maxillary defect was captured using a small-sized stock dentulous tray and irreversible hydrocolloid (Alginate, Ruthinium Dental Products Pvt. Ltd., Valsad, India). A facial moulage was made by boxing the face with hard modeling wax (Dental Products of India, Chennai, India) and then applying the hydrocolloid. The impression was reinforced with dental plaster to provide support during retrieval and the pouring of the cast. The impressions were poured in Type III Dental Stone (Goldstone, Asian Chemicals, Rajkot, India) (Figure 2).

Border molding was performed using a green stick (DPI Pinnacle Tracing Sticks, Dental Products of India, Chennai, India), and the secondary impressions were made with light-body addition silicone (Variotime Light Flow, Heraeus Kulzer GmbH, Hanau, Germany). The working cast was trimmed, defect undercuts blocked out, and a tinfoil substitute (Acryton Cold Mould Seal Separating Medium, Orthoplast, Khurja, India) was applied.

Fabrication of palatal obturator without artificial teeth

**Figure 3:**
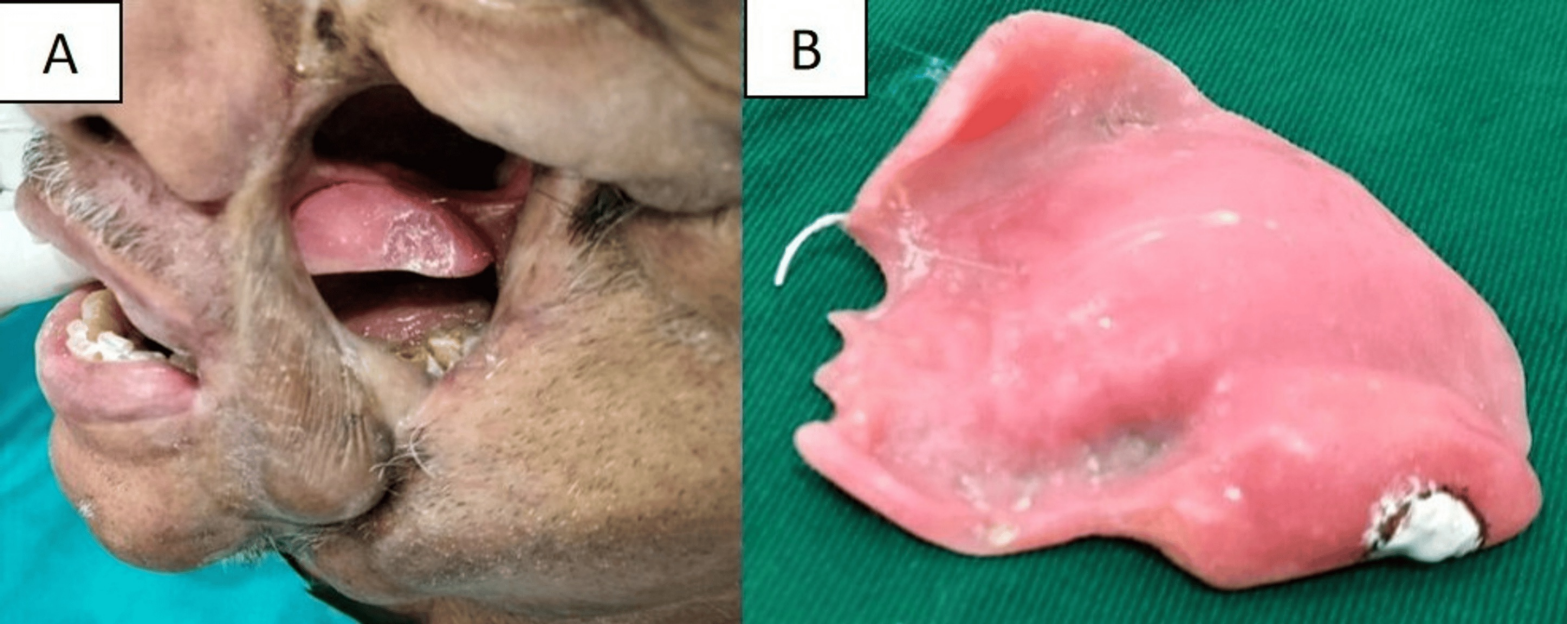
Try-in and insertion of the palatal obturator without artificial teeth. (A) Try-in for the palatal obturator. (B) Acrylized palatal obturator prosthesis without artificial teeth.

A palatal obturator was fabricated using autopolymerizing acrylic resin (RR Cold Cure, Dental Products of India, Chennai, India), without incorporating artificial teeth, and was extended into the left buccal sulcus to seal the defect. The try-in was then performed. A stainless steel Acker's clasp was placed on tooth 13, and the obturator was subsequently acrylized in heat-polymerizing acrylic resin (Heat Cure, Dental Products of India, Chennai, India) using conventional techniques for removable denture fabrication (Figure 3).

Fabrication of cheek prosthesis

**Figure 4:**
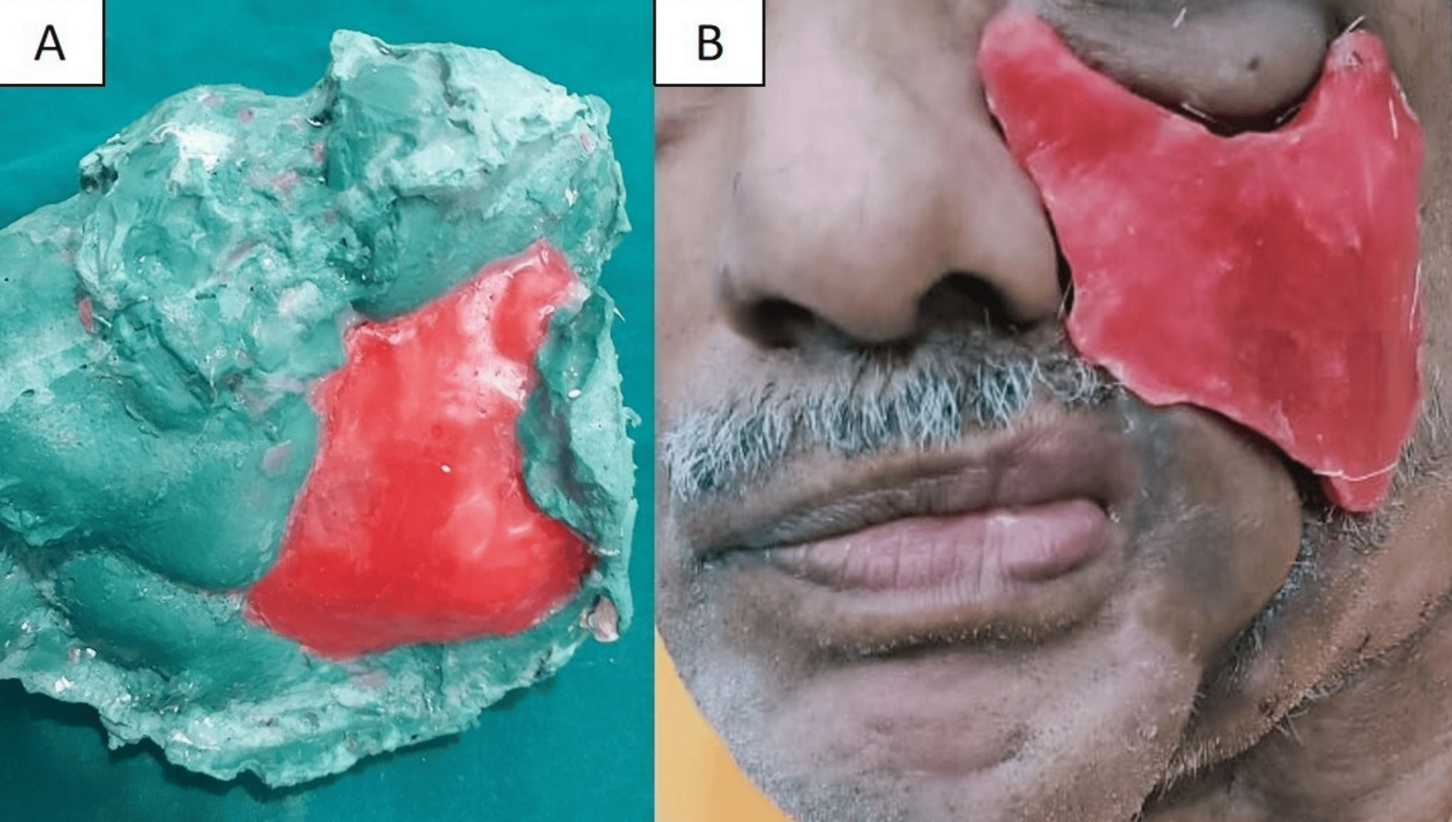
Wax pattern and try-in of the cheek prosthesis on the mid-facial defect. (A, B) Wax pattern for cheek prosthesis created, contoured and adjusted.

A wax pattern for the cheek prosthesis was created on the cast, contoured, and adjusted on the patient's face (Figure 4).

**Figure 5:**
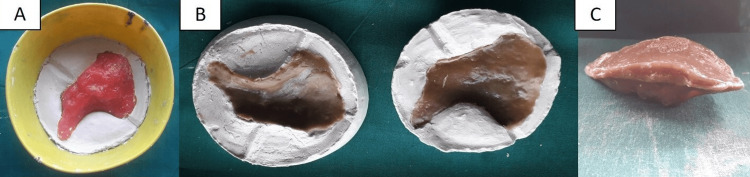
Fabrication of a single hollow cheek prosthesis. (A) Wax pattern invested in dental plaster. (B) Double plaster molds and cheek prosthesis. (C) Single hollow cheek prosthesis.

The cheek prosthesis was fabricated in two parts (inner tissue surface and outer external surface), with double plaster molds. The molds were created using a rubber bowl, incorporating each half of the wax pattern into the plaster, with grooves on each part to ensure accurate assembly. Acrylic pigments (Fevicryl Acrylic Colors, Pidilite Industries, Mumbai, India) were incorporated into the autopolymerizing acrylic resin to match the patient's skin tone. The two parts were joined with the resin to form a single hollow cheek prosthesis (Figure 5).

Insertion of the prosthesis

**Figure 6:**
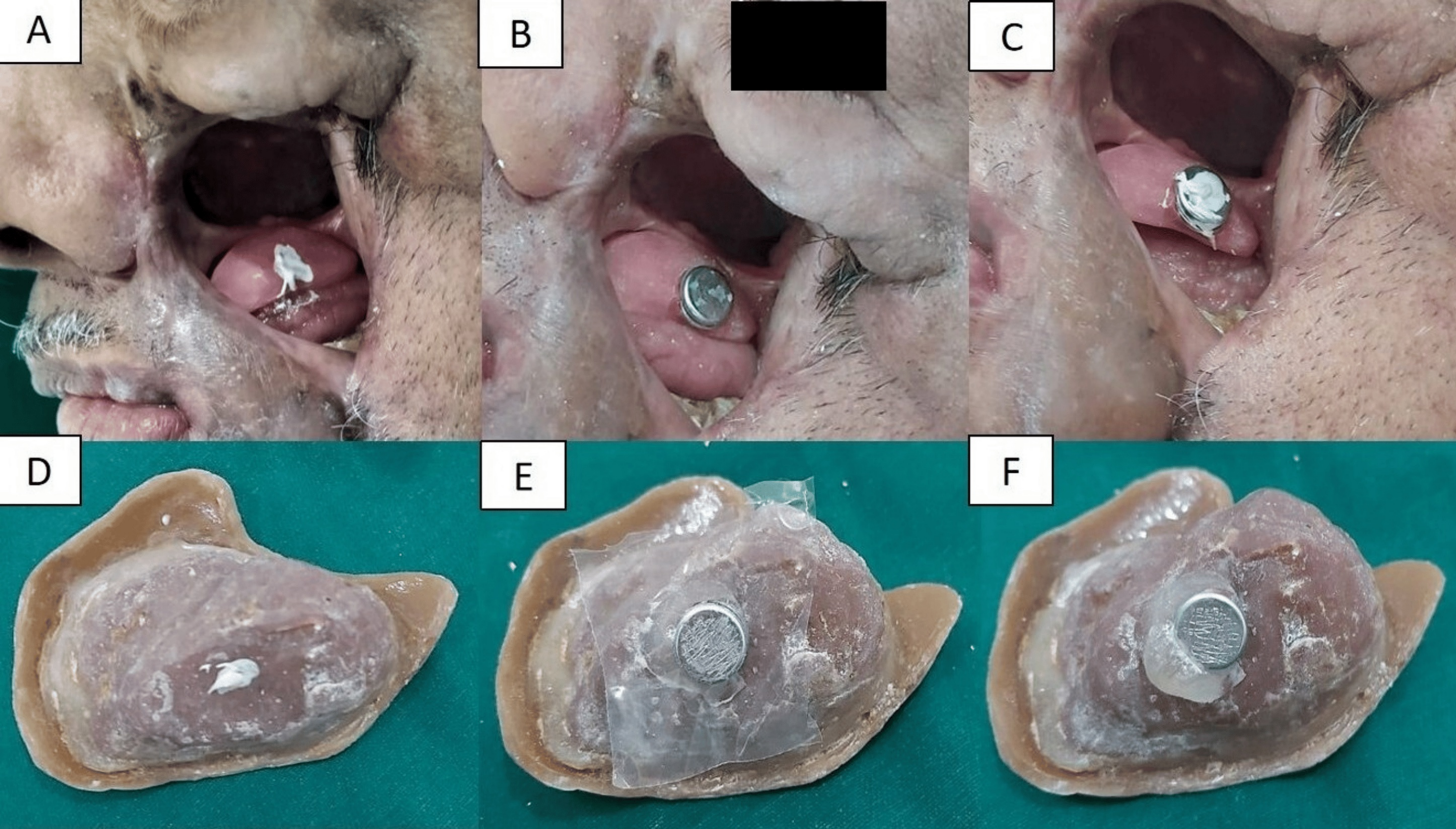
Locating the magnetic assembly using pressure indicating paste on the palatal obturator and cheek prosthesis. (A) Pressure-indicating paste on the palatal obturator to locate magnetic placement. (B) Magnetic assembly attached to the buccal side of the palatal obturator. (C) Pressure-indicating paste on the palatal obturator to locate cheek prosthesis placement. (D) Cheek prosthesis with pressure-indicating paste to locate magnetic placement. (E, F) Attaching the magnetic keeper on the cheek prosthesis with autopolymerizing acrylic resin.

The palatal obturator without artificial teeth was inserted. Pressure-indicating paste was applied to locate the optimal placement for a 1.5-mm-thick, 2-mm-diameter cobalt-samarium (Co5Sm) magnet (Jobmasters, Randallstown, USA). The paste’s location was transferred from the palatal obturator to the cheek prosthesis. Magnetic assembly was placed on the buccal side of the palatal obturator, and a keeper was fixed into the cheek prosthesis with autopolymerizing acrylic resin. The magnet housings were roughened for mechanical retention (Figure 6).

**Figure 7:**
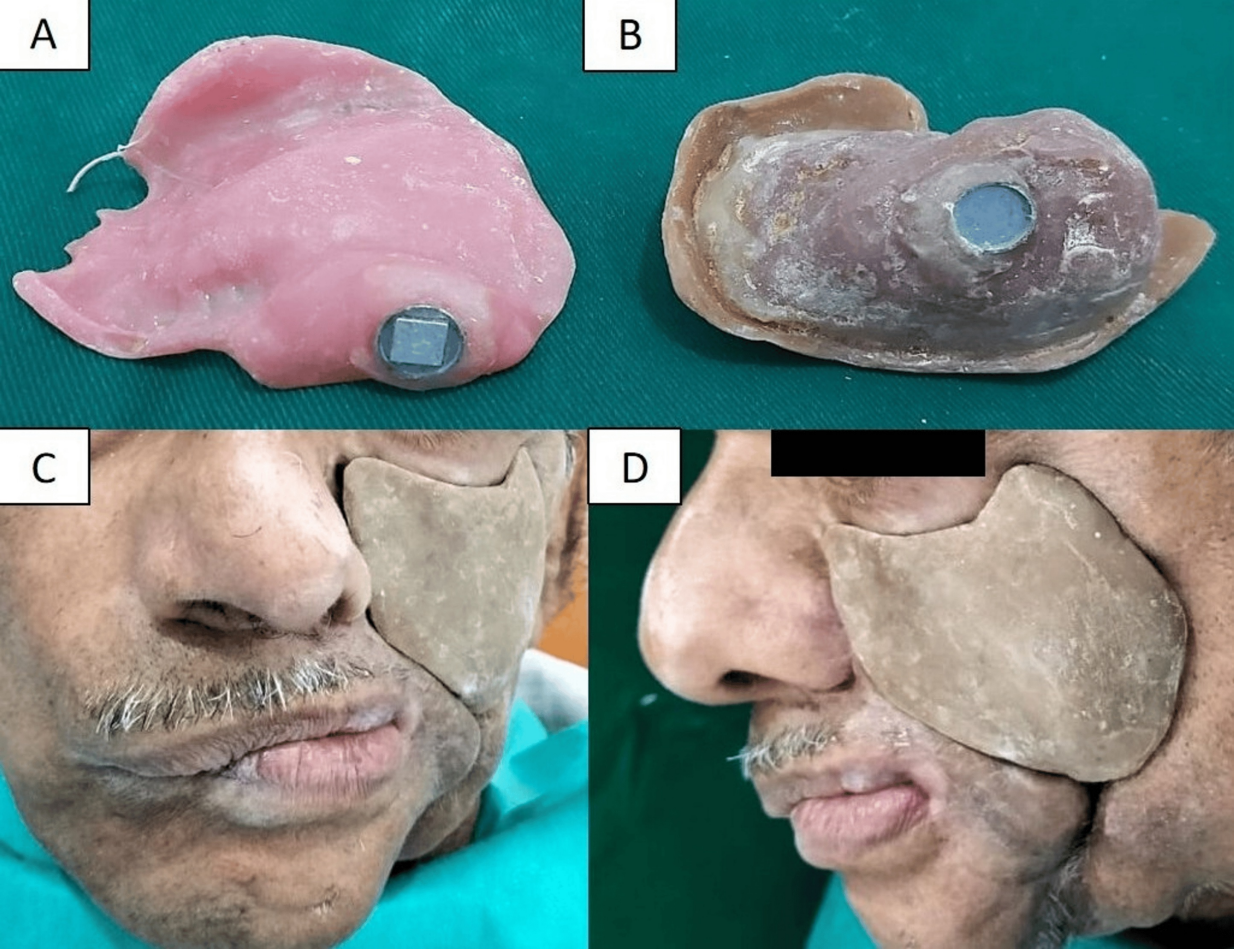
Prosthetic rehabilitation of the mid-facial defect using an interim magnet-retained two-piece acrylic prosthesis. (A) Palatal obturator without artificial teeth incorporating the magnetic assembly. (B) Cheek prosthesis with magnetic keeper. (C) Extraoral frontal and (D) lateral view of the prosthetic rehabilitation.

Both prostheses were trimmed to remove over-extensions, assembled separately, and inserted in place using magnetic retention (Figure 7).

Post-insertion and follow up

The patient was asked to eat semi-solid food using a spoon, drink water, and instructed to pronounce high-pressure sounds (‘f’ and ‘s’) to assess prosthesis function [8]. The patient was satisfied with the prosthesis in relation to most of the concerns; however, mastication and speech were affected. Post-insertion prosthesis and oral hygiene maintenance included cleaning using a soft-bristled toothbrush and toothpaste. Immersion of the prosthesis in 4% chlorhexidine for one minute, followed by rinsing with water was also recommended. The patient also was instructed to use 0.12% chlorhexidine mouthwash once daily. Proper handling and insertion/removal techniques were advised. The patient was instructed to avoid excessive force to protect the magnetic system. A follow-up was scheduled for the next day, with monthly recall visits planned for up to six months. The patient meticulously followed the instructions, but adjustments were needed due to fibrotic tissue healing, requiring relining with autopolymerizing acrylic resin.

## Discussion

A multidisciplinary approach is essential for the rehabilitation of complex mid-facial defects [9,10]. In this reported case, the complications and delayed prosthetic rehabilitation led to the fabrication of a palatal obturator without artificial teeth, which adversely affected both masticatory and speech outcomes during rehabilitation [11].

The cheek prosthesis was hollowed to reduce weight. Acrylic was chosen for its versatility, allowing for trimming and relining while achieving optimal outcomes. Several retention methods, such as spectacles, adhesives, osseointegrated implants, and magnets, have been discussed in the literature [12,13]. Although implants provide reliable retention, further surgery was contraindicated. Adhesives and spectacles were rejected due to questionable long-term retention and the patient’s preference for a prosthesis without visible defects when spectacles were removed. Therefore, magnets were selected as the most suitable option to retain the prosthesis. Border molding and secondary impressions were performed to secure the peripheral seal, maximizing anatomical support and retention from the defect undercuts. Additionally, the Acker's clasp placed on tooth 13 retained the palatal obturator without artificial teeth.

Magnets have been used for retention in overdentures, maxillofacial prostheses, and removable partial and implant-supported dentures [14]. Nadeau was the first to apply magnets in maxillofacial prostheses, using them to connect extra- and intraoral prostheses [15]. Later, Federick introduced an interim magnetic retained maxillary obturator [16]. Robinson developed a two-section intraoral prosthesis using magnets for obturator [17]. Strnat K et al. introduced the Co5Sm magnet alloy [18]. Sasaki H et al. reported its successful use for retaining sectional prostheses in microstomia patients [19]. These rare earth-coated magnets are preferred for their strength, durability, heat and corrosion resistance, and for being simple, low-cost, self-adjusting, and tissue-friendly [20]. In this case, Co5Sm magnets were placed on each component of the two-piece prosthesis, with opposite poles facing each other. The small size and concealed nature of the magnet made the prosthesis aesthetically acceptable, thereby reducing the psychological burden of wearing it [20]. Magnetic forces provided excellent retention, preventing prosthesis displacement during function and ensuring easy insertion and removal. The magnet’s ability to dissipate lateral forces also helped protect the surrounding tissues. While magnets may lose retention over time, they can be easily repaired and reset [20].

## Conclusions

The interim, magnet-retained, two-piece acrylic prosthesis rehabilitated the complex mid-facial defect, which satisfactorily addressed most of the patient’s chief concerns. The cheek prosthesis improved facial appearance, while the palatal obturator sealed the intraoral defect, enhancing swallowing and preventing nasal regurgitation and oral fluid leakage; however, mastication and speech were affected. The patient continued with a semi-solid diet and was able to masticate using the remaining dentition. The palatal obturator slightly improved speech by sealing the defect, although slurring persisted. The combined anatomical support and magnetic retention enhanced the retention, stability, and support of the prosthesis. This non-invasive and cost-effective solution was tailored to meet the patient’s specific needs and limitations. As an interim prosthesis, it effectively fulfilled its rehabilitative purpose, even after six months.
